# Qualitative assessment of the national initiative to implement antimicrobial stewardship centres in French administrative regions

**DOI:** 10.1186/s13756-023-01245-9

**Published:** 2023-04-25

**Authors:** Michèle Conlin, Anne-Gaëlle Leroy, Antoine Asquier-Khati, David Boutoille, Gabriel Birgand

**Affiliations:** 1 National center for the surveillance and prevention of the antimicrobial resistance and healthcare associated infections in primary cares and nursing homes, PRIMO, France; 2 Service des Maladies Infectieuses et Tropicales, CIC-UIC 1413 INSERM, Centre Hospitalier Universitaire, Nantes, France; 3 Centre d’appui à la prévention des infections associées aux soins des Pays de la Loire, 5 rue Pr Yves Boquien, Nantes, 44093 France; 4 NIHR Health Protection Research Unit, Antimicrobial Resistance and Healthcare Associated Infection at Imperial College London, Hammersmith Campus, London, W12 0NN UK

**Keywords:** Health Policy, Antimicrobial stewardship, Microbial Drug Resistance, Implementation

## Abstract

**Background:**

In May 2020, the French Ministry of Health funded the creation of regional antimicrobial stewardship (AMS) coordination centres (CRAtb) in preparation for the new national framework for the prevention of antimicrobial resistance. This study aimed to assess through qualitative methods the implementation process, the activities carried out, and the interactions with other regional stakeholders of the newly created CRAtb.

**Methods:**

We conducted a mixed-method study based on a cross-sectional survey and semi-structured interviews by French regions among implemented CRAtb. Of the eight eligible French regions with an existing CRAtb, seven participated to the online survey. Regional partners involved in AMS from the eight regions were interviewed between September 2021 and April 2022. The survey questionnaire addressed, through closed questions, the organization of the CRAtb, articulation with other regional actors involved in AMS and infection prevention and control (IPC), and AMS activities. The semi-structured interviews approached the implementation and the role of CRAtb, and the collaboration of other AMS and IPC stakeholders. Interview transcripts were analysed using thematic content analysis methodology.

**Results:**

AMS activities carried out by CRAtb were mainly focusing on hospitals (n = 3), primary care (n = 2) and nursing homes (n = 1). Education mostly relied on training days and AMS help lines, communication on websites and newsletters. CRAtb members reported still being more engaged in providing advice to professionals for individual antibiotic treatments rather than collective-level AMS activities. Interactions were frequent between CRAtb, IPC regional centres and health authorities, but rarely involved other stakeholders. Interviews were performed with 28 professionals involved in AMS from eight regions. Pre-existing networks and working relationships in AMS and more broadly facilitated the implementation of CRAtb. Streamlining and decompartmentalizing IPC and AMS regional activities were considered a way to optimise the prevention of antimicrobial resistance across sectors. The engagement with liberal health professionals was identified as a significant obstacle for CRAtb.

**Conclusions:**

Two years after the launch of a new national framework, the implementation of CRAtb appeared complex in most regions. An integrative model joining IPC and AMS efforts, relying on existing networks, with engagement from liberal health profession organisations may be the next pivotal step.

**Supplementary Information:**

The online version contains supplementary material available at 10.1186/s13756-023-01245-9.

## Background

Antimicrobial resistance (AMR) is identified by the World Health Organization (WHO) as one of the biggest threats to global public health [1]. The relationship between antibiotic consumption and the rise of AMR has been clearly documented for some time, [2] paving the way for antimicrobial stewardship (AMS) initiatives worldwide. Despite three successive national antibiotic action plans (2001, 2007, and 2011), recent data from the European Centre for Disease Prevention and Control (ECDC) placed France as the fourth largest consumer of antibiotics in primary care in Europe, with 18.7 DDD per 1000 inhabitants in 2020 [3] and 80% of all antibiotic prescriptions originating from this setting [4]. Responding to the critical need to re-examine its national AMS policies, a new French AMR prevention strategy was introduced in 2022 which incorporated, on the one hand, a renewed focus on the primary care sector, and on the other hand, a closer integration of IPC and AMS activities [5].

In France, the public administrative body in charge of regional implementation of the health policies (the Regional Health Agency), is responsible for ensuring proper coordination between the various regional stakeholders [6]. In 2017, regional Infection Prevention and Control (IPC) coordination centres were created to provide expertise and to coordinate regional strategies in healthcare-associated infection prevention [7]. In May 2020, the French Ministry of Health funded, in building its new national framework, the creation of regional centres for the coordination of AMS (CRAtb) with a focus on hospitals, nursing homes, and primary care [8]. Composed at least of an Infectious Disease Specialist and a general practitioner (GP), CRAtb have been tasked with: (i) a mission of expertise and support to health professionals in the field of AMS, and (ii) a mission of coordination of networks of health professionals in charge of AMS programs [9]. The mandate provided a set of guidelines for CRAtb including specific tasks to achieve their missions (e.g. identify regional issues around antibiotic misuse, support the implementation of training policy (initial and ongoing) for health professionals, setup of a tele-expertise service providing advice on antibiotic therapy, among others). In certain regions, CRAtb were intended to replace and build upon pre-existing regional centers for advice on antibiotic therapy. The Ministerial directions outlined a regional strategy position with a collective focus for CRAtb, supported by close collaborations with corresponding regional IPC teams. The national framework further included the creation of locally operating AMS and IPC consultant teams, aiming to provide on call or onsite advice to local stakeholders.

In this study, we conducted a qualitative evaluation to better understand how CRAtb were implemented in French regions following the national initiative, the interventions carried out, partnerships and collaborative efforts.

## Methods

### Study design and setting

We conducted a qualitative case study analysis to assess the implementation of the CRAtb in French regions. A cross-sectional survey using an online questionnaire, and semi-structured interviews were conducted to answer three research questions: (1) What resources and activities CRAtb engaged two years following the launch of the national initiative? (2) How did regional IPC, AMS and health authority’s partners describe the barriers and facilitators of CRAtb implementation? (3) How did the different partners describe the alignment and coordination of the AMS efforts in their region? The study included CRAtb officially created at the time of the study corresponding to eight of the 17 eligible French regions.

### Data collection and analysis

Between September 2021 and April 2022, active members from the eight existing CRAtb were invited to answer an internet-based survey addressing (i) human resources, (ii) implemented activities (education and training, communication, monitoring and feedback, evaluation and audit), and (iii) interactions (frequency of meetings) with other regional actors involved in the prevention of AMR. (Supplemental Document S1) In addition to the online questionnaire, CRAtb and their corresponding regional IPC coordinating centres and Regional Health Agency from the eight regions were contacted by e-mail and invited to participate in semi-structured interviews. All the eight regions contacted agreed to participate and completed a one-time videoconference interview between February 2022 and April 2022.

A thematic interview guide (Table 1) was developed by the research team and pilot tested to ensure clarity and comprehensiveness. The guide focused on three areas: implementation of CRAtb, the role of CRAtb in AMR prevention, and interactions between AMR prevention stakeholders. The full interview guide was used with CRAtb coordinators, however only the third section on focusing on interactions between stakeholders was used during interviews with regional IPC coordinating centre and Regional Health Agency representatives. Interviews were carried out by three researchers (AA, AGL, MC). All of the interviews were done through videoconferencing, recorded in their entirety, transcribed verbatim, and anonymized prior to analysis. Interviews were carried out in French. Interview transcripts were analysed using thematic content analysis methodology [10]. Both deductive and inductive coding was used, with an *a priori* codebook developed from the interview guide (Supplemental Table S2). Analysis of transcripts was conducted by two researchers (AGL, MC) in parallel after having jointly analysed two transcripts for coding adequacy and consistency. Once parallel coding was complete, the researchers reviewed the results to discuss any coding inconsistencies and to identify major, minor, and cross-cutting themes. QDA Miner Lite software (Version 2.0.9; Provalis Research, Montreal, Canada) was used to facilitate data analysis. Supporting quotes were translated into English by MC (native English/French speaker) and reviewed for accuracy by AGL and GB (native French/fluent English speakers).

**Table 1:** Semi-structured interview guide

**Position and role in the CRAtb**	What position do you hold in your CRAtb? How did you get to the position you hold today?
**Implementation of the CRAtb**	• Who were the actors involved in the prevention of antibiotic resistance before the establishment of the CRAtb in your region? • When the CRAtb was established, how did you work with other regional actors involved in the prevention of antibiotic resistance? Please give examples. • When the CRAtb was established, how did you work with other regional actors involved in the prevention of antibiotic resistance? Please give examples. • What were the biggest obstacles to the establishment of the CRAtb*?* • What positive changes have occurred with the establishment of the CRAtb in your region?
**Role of the CRAtb in the regional coordination of antibiotic resistance prevention**	• What are the activities of your CRAtb for the regional coordination of the prevention of antibiotic resistance? • In your opinion, which of your activities are most critical to the prevention of antibiotic resistance? • What are the greatest strengths of your CRAtb in preventing antibiotic resistance in your region? • What are your CRAtb’s weaknesses in preventing antibiotic resistance in your region? Please give examples. How could these weaknesses be improved? • What are the main actions to come in the next few months? Please specify.
**Interactions with other regional antibiotic resistance prevention stakeholders**	• In your region, how does the CRAtb work with the various actors in the prevention of antibiotic resistance? Please give examples. • How does the CRAtb communicate with other actors involved in antibiotic resistance prevention? How is data shared? • How would you describe the relationship between your CRAtb and other stakeholders involved in antibiotic resistance prevention? How could they be improved? Are there future collaborations to come? • If there are no interactions with some of the actors in the prevention of antibiotic resistance, can you explain why? Can you comment on possible future collaborations? How could this be approached?
**Final question**	Is there anything we haven’t talked about that you think is important to address?

Abbreviations: CRAtb, regional antimicrobial stewardship coordination centres

## Results

**Fig. 1:**
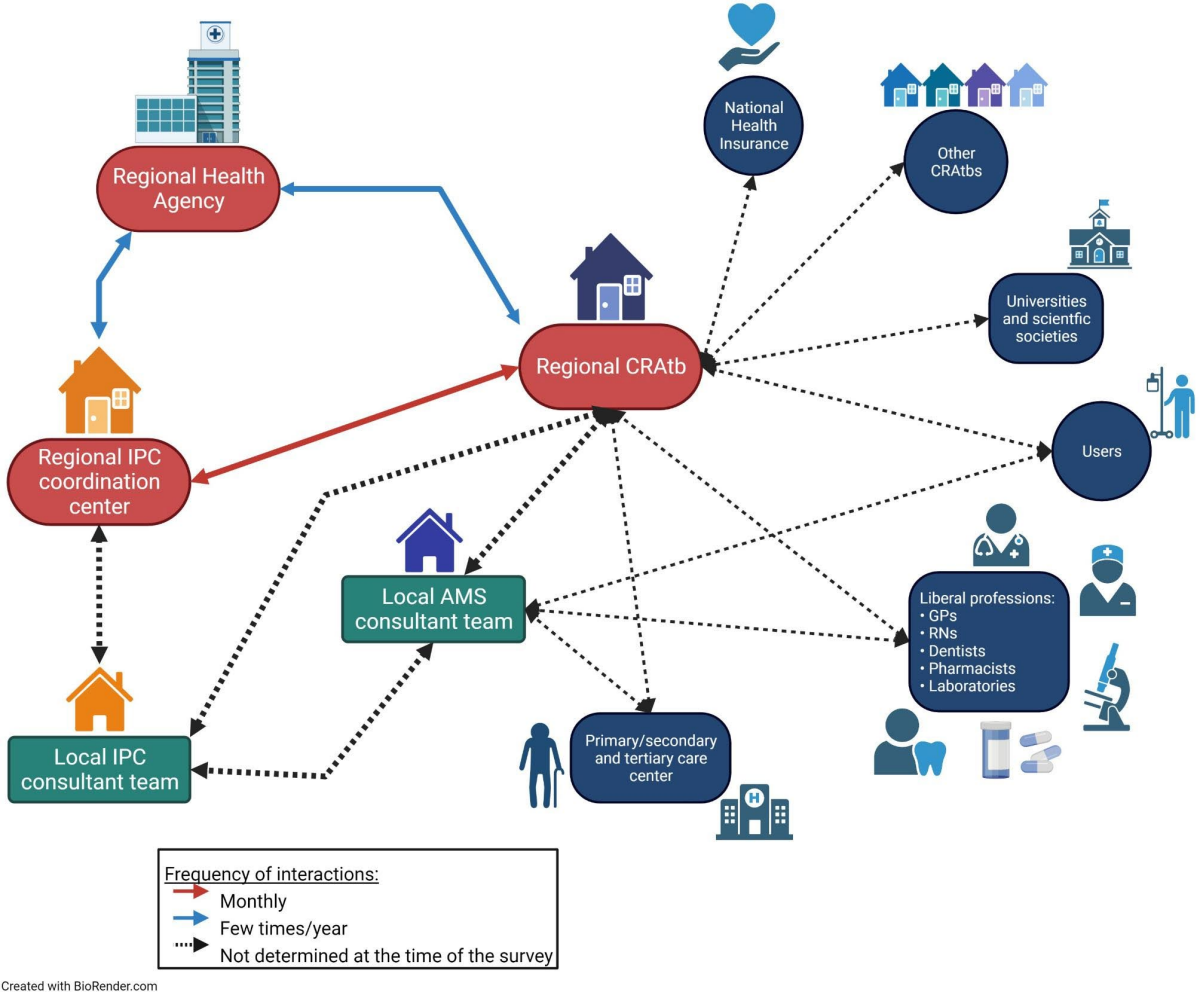
Description of collaborations engaged by the CRAtb with the actors involved in the prevention of antibiotic resistance Abbreviations: CRAtb, regional antimicrobial stewardship coordination centres; AMS, Antimicrobial Stewardship; IPC, Infection Prevention and Control; GP, General Practitioner; RN, Registered Nurse

Of the eight CRAtb contacted, seven responded to the online survey. The official creation dates of the participating CRAtb ranged from 2020 to 2022, with structures therefore at different stages of implementation. (Table 2) In accordance with the national framework, Infectious Disease Specialists and GPs were systematically engaged in all CRAtb, mostly part time. Five CRAtb (over 7) involved other professional categories such as clinical microbiologists (n = 3, 0,2 to 0,7 FTE), pharmacists (n = 3, 0,2 to 0,7 FTE), infection prevention and control specialist (n = 1, 0,7 FTE), public health specialist (n = 1, 0,4 FTE), and engineer (n = 1, 0,2 FTE). Among the six CRAtb that answered to the question pertaining to which care sector was currently the focus of most of their actions, three declared focusing on AMS in hospitals, two on primary care, and one on nursing homes. Training days on AMS were organised by all CRAtb and AMS help lines were open in six. Among communication tools, six CRAtb owned a website and four shared newsletters. The frequencies of interactions between the main regional partners involved in AMS are presented in Fig. 1. CRAtb mainly organised frequent meetings with health authorities (monthly in one region and quarterly of more for others) IPC regional centres (monthly in two regions and quarterly of more for others). Interactions with other stakeholders (e.g. local AMS actors, health insurance, users) were infrequent to inexistent.

**Table 2:** Description of human resources, collaborations and AMS activities implemented by the seven CRAtb that participated to the survey

	Regions/CRAtb							
Region number	1	2	3	4	5	6	7	
Creation date	2021	2020	2020	2022	2021	2022	2021	
**Human resources** (**FTE)**								
Infectious Disease Specialist	1.4	0.5	0.3	0.7	0.5	0.8	0.5	
General Practitioner	0.5	0.2	0.8	0.7	0.1	0.4	1	
Other specialties*			0.5	0.7	0.4	0.4	0.8	
**AMS Activities**								
Main focus of activities	H	-	H	PC	H	PC	NH	
*Education for health professionals*								
Training days	**×**	**×**	**×**	**×**	**×**	**×**	**×**	
AMS helpline		**×**	**×**	**×**	**×**	**×**	**×**	
Webinars		**×**	**×**	**×**	**×**		**×**	
Quick reference prescribing guides		**×**	**×**	**×**		**×**	**×**	
e-Learning	**×**		**×**			**×**	**×**	
Tele-expertise							**×**	
*Communication tools*								
Website		**×**	**×**	**×**	**×**	**×**	**×**	
Newsletters			**×**	**×**		**×**	**×**	
Social networks			**×**			**×**		
Smartphone applications			**×**					
*Other AMS activities*								
AMS activities aimed at the general public			**×**	**×**	**×**	**×**	**×**	
Use of AMS indicators	NH	-	H	H	H	H	NH	
Monitoring system for implemented activities	**×**				**×**	**×**	**×**	
Practice audits/ activity evaluation		**×**	**×**			**×**	**×**	
Research			**×**	**×**	**×**	**×**	**×**	

Legend : ×, activity performed

Abbreviations: AMS, Antimicrobial stewardship; IPC, Infection Prevention and Control; FTE, Full-time equivalents; H, hospital, NH, nursing home, PC, primary care

Footnote: *others medical specialties: clinical microbiologists (n = 3), pharmacists (n = 3), IPC specialists (n = 1), public health specialists (n = 1), engineer (n = 1)

A total of 21 interviews were held with 28 participants from CRAtb (n = 10 participants), IPC coordinating centres (n = 9), and Regional Health Agencies (n = 9). Details of participants are available in Supplemental Table S3. Fifteen interviews were one-on-one, though at the request of participants, six interviews comprised two or three participants with shared roles in the same structure. Interviews lasted between 10 and 50 min.

Stakeholders involved in CRAtb implementation were mostly wrestling with the challenges of adapting to a new operational organization and applying the national framework within regional and local realities. Three cross-cutting themes were identified in the semi-structured interviews which related to a spectrum of implementation aspects: (i) streamlining and decompartmentalizing IPC and AMS activities, (ii) engaging with liberal health professionals (i.e., those working outside of public healthcare establishments, namely GPs, primary care nurses, and community pharmacists and dentists), and (iii) the role of pre-existing networks and working relationships. (Table 3, Supplemental Table S4)

**Table 3:** Identified themes and illustrative quotations

Cross-cutting themes
T1- Streamlining and decompartmentalizing IPC and AMS activities *Related codes: Planning, implementation facilitators, CRATB strengths, interaction facilitators*
Subtheme: shared human and material resources Q1: "But actually we [CRATB and IPC coordinating center] have our offices in the same place. We have our offices, really close together. So it’s pretty easy to interact.” CRATB coordinator (Region 4) Q2: “We have the same offices, we have shared staff, shared secretarial support, we have a shared practitioner because we have a practitioner who is half-time [IPC coordinating center], half-time [CRATB]." IPC coordinating center coordinator (Region 1) Q3: "That is to say, she [the CRATB research engineer] has two positions. 80% on the [IPC coordinating center] mission and 20% on the CRATB. But it isn’t a person who is within one entity who’s made available for the other. And the secretary is the same. The secretary is a full-time employee of the [IPC coordinating center] who, on an ad hoc basis, provides assistance to the CRATB. For example, for mailings, sending out documents. Things like that, providing mailing lists for emails, for communication.” IPC coordinating center coordinator (Region 3) Q4: "So we have the mailing lists, we have [IPC coordinating center] data and data from other missions on [AMR] that are, that go through the [IPC coordinating center] and that are sent to the CRATB, for example." IPC coordinating center coordinator (Region 3) Subtheme: Effective collaboration Q5: "And so I think that the [locally operating IPC consultant teams], when they are set up, and the [locally operating AMS consultant teams], will have every interest in working together since... it won’t always be the same actors, but still Infectious Disease Specialists, coordinating physicians, GPs, pharmacists, and GPs. Nurses eventually as well. So it is in our interest to work together to intervene in nursing homes and make things evolve in terms of [AMS] and infection prevention. So we have everything to gain by working together" CRATB coordinator (Region 1) Q6: "We are really, sincerely, the key words, it’s really to decompartmentalize. We’re trying to decompartmentalize and to mutually call upon each other as soon as we do workshops, as soon as... even if they are fields that, a priori, do not directly concern either the [IPC coordinating center] or the CRATB." IPC coordinating center coordinator (Region 4) Q7: "We have [X number] of mobile [IPC] teams that cover the region and it’s clear that we need to ensure that they fit into this organizational chart for [AMS]. They are all quite enthusiastic about it, most of them are very, very keen on activities to promote [AMS] in nursing homes, which will probably be the key interface to reach the liberal sector.” IPC coordinating center coordinator 2 (Region 5) Q8: "Well, there are two circles. There’s the [IPC coordinating center] agenda and the CRATB agenda. There’s a common core to both actually. They connect on parts of their agendas." Regional Health Agency officer (Region 4) Q9: "Or when there’s a problem, for example, when we’re asked to give advice. This happens to me from time to time in nursing homes. As soon as the situation drifts towards treatment, curative care, we hand over to the Infectious Disease Specialist... We hand over to the Infectious Disease Specialist because each of us has our own area of expertise. And that’s it. I regularly call on our colleagues here [Hospital in city X], who are the [locally operating AMS consultant team], for advice on antibiotic therapy. That’s pretty comfortable, actually." IPC coordinating center coordinator (Region 3)
T2- Engaging with liberal health professions *Related codes: Engaging, implementation barriers, CRATB strengths, CRATB weaknesses, interaction facilitators, interaction barriers*
Subtheme: Hiring GPs for CRATBs and locally operating AMS consultant teams Q1: "It was a hurdle [hiring a GP] for several months. Clearly, I spent a lot of time on it. It took... I contacted over a hundred GPs myself by getting them on the phone. Contacted them by email." CRATB coordinator (Region 4) Q2: "I And do you have any idea why it was difficult to motivate GPs to join this type of structure? P Perhaps first of all, as the GP contact network wasn’t extremely complete, at least as far as I was concerned, perhaps the information wasn’t circulated enough. And then, we have funding for a part-time GP. With the budget we had at the beginning, which was miserable, we had no chance of being able to convince a GP to join us part-time with the pay we were going to offer him. It took two months to be able to get a salary increase. It’s just hard to recruit a part-time GP like that.” CRATB coordinator 1 (Region 5) Subtheme: Communication and reach towards liberal professions Q3: "It’s true that if we can’t find a GP, if we can’t reach them, if... there’ s perhaps a point... The weakness is perhaps a difficulty in reaching the liberal world. By its [the CRATB] organization, already, from the outset by its organization in particular, carried by a university hospital, that’s perhaps its weakness.” CRATB coordinator (Region 7) Q4: "So, in order to have an impact on [primary care], it will be really interesting. In addition to having three [GP] colleagues who can help us... who can think and help us reach out to that sector." CRATB coordinator 1 (Region 3) Q5: "And our second hope is really to have a tool to act on [primary care] and to collaborate a little bit better with [the primary care sector]. We have a link, we have this hotline, so we’ve had a fan club of GPs for a long time. But on the other hand, we don’t reach, or with great difficulty, a large part of GPs who don’t ask for advice and work alone. Now I have hope that this will give us tools.” CRATB coordinator 2 (Region 5) Q6: "Where we have, I find, we have a lot of... a lot of room for improvement, is with respect to [primary care], [primary care] prescriptions. And that we haven’t tackled because we have… it’s difficult to reach them directly. We have to go through the [Regional Union of Health Professionals]. And then, if we don’t have specific information, we never get the mailing lists so we have to give them the information. And then if it works... Well, it’s then distributed to all the liberal physicians, but if not, we can’t." IPC coordinating center officer 1 (Region 5) Q7: "I don’t know how many thousands of GPs we have in [Region 3]. We don’t have an easy entry point. It’s very complicated. [...] Through the COVID crisis, we had some connections with [primary care], but I won’t lie to you, it’s not great, the [primary care sector]. Why isn’t it great? Because actually, the entry points are complex [...]." IPC coordinating center coordinator (Region 1)
T3- Role of pre-existing networks and working relationships *Related codes: Planning, relationships, engaging, implementation facilitators, implementation barriers, CRATB strengths, interaction facilitators*
Q1: "Because in [Region 2] we can almost say that the CRATB has existed informally for 10 years. What I mean is that we are really in contact with many stakeholders from different catchment areas and different specialties. So there is a certain organization that’s already there.” CRATB coordinator (Region 2) Q2: "Well, its strengths, as I said earlier, is having existing resources, practitioners and... who know each other, local actors who have already worked together and projects that have already been developed and are running, particularly in terms of training. That, I think, is one of our great assets.” CRATB coordinator 1 (Region 3) Q3: "The strong points I think is the history, since [the previous AMS structure] exists since 2003. So recognized for a while. I mean [the AMS network] is a network that is starting to be known in the region. So it gives some weight. And then the doctors call for telephone advice. They come for conferences, consult the website even if it’s not... It could be better. But, so... There is still a history, a recognition at the regional level, collaborations with many infectious disease services and structures in the region.” CRATB coordinator (Region 1) Subtheme: Continuity of relationships and actions Q4: "Because now, we’re thinking that the CRATB will promote a lot of the actions that we were carrying out before with [the previous AMS structure] in order to get our foot in the door.” CRATB coordinator (Region 8) Q5: "We have to build our regional organization in relation to the previous one. We’re not going to say, let’s just sweep up everything and start over and... that’s pointless." Regional Health Agency officer (Region 4) Q6: "The CRATB is being established. And we change nothing. In fact, we are continuing the actions that are already in place. We will, we will, we won’t change anything, we’ll continue these actions and we’ll especially develop new ones because we’re better structured.” CRATB coordinator 2 (Region 3)

CRATB: Regional antimicrobial stewardship coordination centers; IPC: Infection Prevention and Control; AMS: Antimicrobial Stewardship

### Streamlining and decompartmentalizing IPC and AMS activities

As outlined in the national framework, CRAtb are expected to collaborate closely with their IPC counterparts. To facilitate this, many teams decided to opt for physical proximity, choosing to share offices with IPC teams. Moreover, CRAtb and IPC coordinating centre teams saw the benefit of pooling their resources through sharing of support staff (secretaries and data managers, for example), as well as sharing of consultants (Infectious Disease or Infection Control Specialists).

*“We have the same offices, we have shared staff, shared secretarial support, we have a shared practitioner because we have a practitioner who is half-time [IPC coordinating center], half-time [CRAtb].“* IPC coordinating center coordinator (Region 1).

With IPC having a well-established history in the French healthcare system, CRAtb were able to further benefit from gaining access to IPC centres’ regional directories and health data.

*“So we have the mailing lists, we have [IPC coordinating center] data and data from other missions on [AMR] that are, that go through the [IPC coordinating center] and that are sent to the CRAtb, for example.“* IPC coordinating center coordinator (Region 3).

Beyond the practicalities of shared human and material resources, participants from all three organizations (Regional Health Agencies, IPC coordinating centres, CRAtb) elicited the advantages of working in close partnership with IPC teams, for example when working with care facilities in which both preventative and curative actions are needed, or in developing workshops and educational material.

*“We are really, sincerely, the key words, it’s really to decompartmentalize. We’re trying to decompartmentalize and to mutually call upon each other as soon as we do workshops, as soon as… even if they are fields that, a priori, do not directly concern either the [IPC coordinating center] or the CRAtb.“* IPC coordinating center coordinator (Region 4).

Building on the complementarity between IPC and AMS in an integrative and sustained way was widely acknowledged as a key factor in a more effective approach to combating AMR.

*“We have [X number] of mobile [IPC] teams that cover the region and it’s clear that we need to ensure that they fit into this organizational chart for [AMS]. They are all quite enthusiastic about it, most of them are very, very keen on activities to promote [AMS] in nursing homes, which will probably be the key interface to reach the liberal sector.”* IPC coordinating center coordinator 2 (Region 5).

*“It is very important because [AMS] cannot be dissociated from infections, in fact, from infection prevention. When, indeed, an action on [AMS] must also include the hygiene component, the diagnostic component… So it’s true that we have to work differently and it has to be instinctive, if I may say so. And in fact, the organization that we want to put in place is designed to promote this sharing and this joint work.”* Regional Health Agency officer (Region 6).

Participants recognized that the boundaries of each specialty overlapped whilst remaining distinct yet complementary in other areas.

### Engaging with liberal health professionals

CRAtb are required to have a GP as a core team member and when possible, as a member of the locally operating AMS consultant teams. However, recruitment of GPs was a difficult process for the majority of CRAtb, and some participants expressed concern about remaining too hospital-centred should they not succeed in obtaining substantial GP participation.

*“It’s true that if we can’t find a GP, if we can’t reach them, if… there’ s perhaps a point… The weakness is perhaps a difficulty in reaching the liberal world. By its [the CRAtb] organization, already, from the outset by its organization in particular, carried by a university hospital, that’s perhaps its weakness.”* CRAtb coordinator (Region 7).

Factors impacting the recruitment process were thought to be financial (modest salary), lack of time, and finding adequate channels for advertising the position. Having a GP on these teams was nonetheless perceived as a pivotal asset to enhance understanding of prescribing practices, gain access to GP networks, and increase the impact of AMS activities in primary and community care.

*“So, in order to have an impact on [primary care], it will be really interesting. In addition to having three [GP] colleagues who can help us… who can think and help us reach out to that sector.“* CRAtb coordinator 1 (Region 3).

*“And our second hope is really to have a tool to act on [primary care] and to collaborate a little bit better with [the primary care sector]. We have a link, we have this hotline, so we’ve had a fan club of GPs for a long time. But on the other hand, we don’t reach, or with great difficulty, a large part of GPs who don’t ask for advice and work alone. Now I have hope that this will give us tools.”* CRAtb coordinator 2 (Region 5).

Overall, not having an “entry point” (notably for communications) to primary care prescribers and other liberal health practitioners was frequently identified as a significant obstacle across regions.

### Role of pre-existing networks and working relationships

In all eight participating regions, a pre-existing AMS network was identified (namely with Regional Health Agencies, regional branches of the National Health Insurance, the Observatories for drugs, medical devices and therapeutic innovations, hospital Infectious Disease teams and Regional Unions of Health Professionals – with variations between regions). These networks and associated working relationships were perceived as defining factors in the implementation of CRAtb. Participants acknowledged the facilitating aspect of having known regional stakeholders who were accustomed to working together. Furthermore, some of the networks were long established, contributing to regional recognition and legitimacy of these actors in engaging in AMS strategies and actions.

*“The strong points I think is the history, since [the previous AMS structure] exists since 2003. So recognized for a while. I mean [the AMS network] is a network that is starting to be known in the region. So it gives some weight. And then the doctors call for telephone advice. They come for conferences, consult the website even if it’s not… It could be better. But, so… There is still a history, a recognition at the regional level, collaborations with many infectious disease services and structures in the region.”* CRAtb coordinator (Region 1).

Many regions were also committed to the continuity of already initiated actions, and some participants perceived the establishment of CRAtb as a continuation of what was already being done but within a new and improved framework.

*“The CRAtb is being established. And we change nothing. In fact, we are continuing the actions that are already in place. We will, we will, we won’t change anything, we’ll continue these actions and we’ll especially develop new ones because we’re better structured.”* CRAtb coordinator 2 (Region 3).

**Fig. 2:**
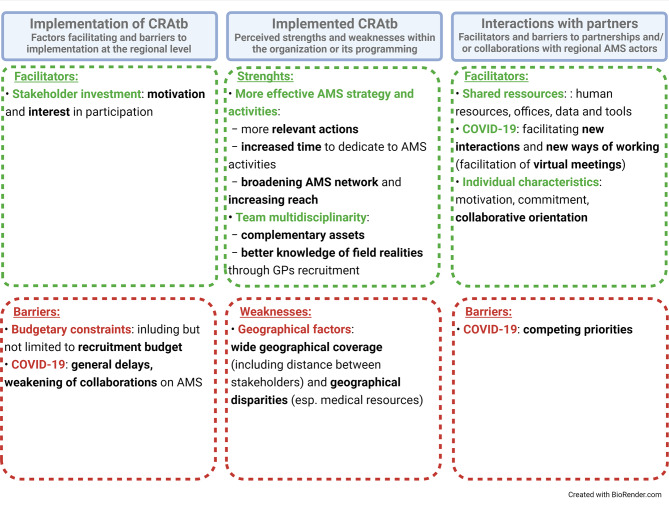
Main themes identified in the semi-structured interviews, relating to the facilitators and barriers to implementation and to partnerships and collaborations, as well to perceived strengths and weaknesses within the CRAtb or its programming (illustrative quotes are found in Supplemental) Abbreviations: CRAtb, regional antimicrobial stewardship coordination centres ; AMS, Antimicrobial Stewardship; IPC, Infection Prevention and Control; GP, General Practitioner

Other themes were identified relating to the facilitators and barriers of implementation and partner interactions, as well to perceived strengths and weaknesses of the CRAtb (Fig. 2, Supplemental Table S4). CRAtb were perceived as a chance to strengthen AMS strategies and activities at the regional level, by the dedicated time allocated to develop relevant actions in an extended network of professionals. However, the geographical region to cover and the disparities appeared as a weakness for the organisation and implementation of consistent AMS actions. The administrative constraints linked to budget use and recruitments was a strong barrier for the implementation of CRAtb, generating delays in recruitments. Interactions with partners involved in AMS were relying on individual motivations and willingness to collaborate. The COVID-19 pandemic provided opportunities for collaborations across stakeholders and specialities, but at the same time appeared as a competing priority with AMS. Finally, most CRAtb participants expressed a strong interest in having regular meetings with other CRAtb to learn from each other’s experiences and share resources. As such, a first national CRAtb meeting was planned during the course of this study.

## Discussion

This study sought to assess the implementation of newly created regional AMS coordination centres in France. The new governance for IPC and AMS is multi-level, relying on the ministry of health providing national priorities and plans, regional centres coordinating the national strategy and actions at the regional level, and local teams providing proximal counselling to healthcare professionals in hospitals, nursing homes, and primary care. This framework aims to harmonize practices at all levels to enhance AMR prevention. At the European level, other countries have implemented national strategies with similar approaches. The Swedish strategic program against AMR (known as Strama), [11] the Scottish management of AMR action plan, [12] and the PIRASOA program (Institutional Programme for the Prevention and Control of Healthcare Associated Infections and Associated Use of Antimicrobial) in Spain’s autonomous community of Andalusia [13] have relied on regional and local groups to adapt national frameworks to local conditions. Key aspects to the success of these European models are also integrated into the French national framework, namely the multidisciplinary nature of regional or local teams, the implementation at multiple levels of care (primary care, nursing homes, hospital), the close collaboration with prescribers, and the linking of IPC with AMS actions [11, 14, 15].

Our findings highlighted the varying level of implementation of CRAtb across regions, with the transition towards the new organizational roles as outlined by the Ministerial directions (with core responsibilities around regional strategies and network coordination) a work in progress. Semi-structured interviews allowed for a more complete picture of the barriers and facilitators to CRAtb implementation, as well as perceived strengths and weaknesses of the model. Our findings suggest that the shift from clinical responsibilities to more collective-focused ones is likely challenging for stakeholders from clinical backgrounds, as evidenced by the continuation of AMS help line activities. The experience of IPC coordinating centres in roles of regional facilitation, coordination, and surveillance may serve as a model for CRAtb coordinators, with the value of collaborating closely and mutualizing resources between IPC and AMS teams clearly acknowledged throughout the qualitative interview data.

With regard to other identified themes in the semi-structured interviews, these are in line with factors involved in effective collaborations in One Health approaches (namely AMR prevention) which have been explored previously [16]. These can serve to inform future implementation efforts in the field and include individual factors (e.g., prior experience and existing relationships), organizational factors (e.g., information sharing, intentional engagement, clearly defined roles), and network factors (e.g., established partnerships, institutionalization of effective collaborative structures). Evaluation in term of implementation and overall impact of these structures were not discussed by participants. Cost-effectiveness analysis of these organisations will be required to better understand the impact of their actions on the antimicrobial use and the antimicrobial resistance.

Building on existing assets is an obvious, valued, and effective approach in developing regional and local AMS strategies (pre-existing organization, stakeholder investment, complementary professional competencies). Being able to engage with liberal health professions working in primary care (such as GPs) is crucial for the prevention of AMR. As mentioned previously, with 80% of antibiotic prescriptions in France stemming from primary care, clear and effective communication channels need to be established with this sector as a priority. This study revealed that for now, having an impact on the primary care sector remained challenging for CRAtb, since half of the structures interviewed reported mostly focusing on AMS in hospitals, and since the difficulties encountered in trying to engage with liberal health professionals were identified as a recurring theme in the semi-structured interviews. IPC and AMS teams have everything to gain by collaborating closely. The ECDC’s proposal for EU guidelines for AMS incorporates this last principle in its recommendations [17]. However, our study demonstrated that French IPC and AMS teams are moving beyond more collaboration and trending towards mergers of operational resources (administrative support, databases and data managers, communication channels). The national strategy’s success may also lie in the facilitation of this approach toward an integrated IPC and AMS model.

This study has a number of limitations. Of note, the research team only interviewed participants from eight out of the 18 French regions. This was due to the current number of formally implemented CRAtb and absence of identified stakeholders in other regions. However, this implies that key issues in the implementation process may have been missed, as those centres which are currently established may have faced less barriers. Further, social desirability bias may be present in the results. Participants may have reported their experiences and perceptions more positively knowing that, though anonymized, their responses would be available to coordinating agencies at the regional and national levels.

## Conclusion

Two years after the publication by the French ministry of health of their legal framework, organisation, and financing, the implementation of CRAtb appeared difficult with the creation of these structures in less than half of French regions. Increased engagement from national and regional liberal health profession organisations is urgently required if advancements in AMS in primary care are to be made. An integrative model of IPC and AMS for the prevention of AMR may be the next pivotal step in addressing this pressing public health threat.

## Electronic supplementary material

Below is the link to the electronic supplementary material.

Supplementary Material 1

Supplementary Material 2

## Data Availability

The datasets used and/or analysed during the current study are available from the corresponding author on reasonable request.
