# Supplementary material for: Qualitative assessment of the national initiative to implement antimicrobial stewardship centres in French administrative regions
Source: Antimicrob Resist Infect Control. 2023 Apr 25;12:41. doi: 10.1186/s13756-023-01245-9 (PMC10127160; doi:10.1186/s13756-023-01245-9)
Supplement: Supplementary file 1 — Supplementary Material 1 [file 13756_2023_1245_MOESM1_ESM.doc]

**Supplementary documents**

**Supplementary document S1. Online survey titled “Overview of the implementation of CRATBs in France”**

**Information on the person completing the questionnaire:**

- Profile of the person filling in the questionnaire
- E-mail address
- Name and first name
- Speciality
- Region
- Institution of affiliation
- Year of beginning of practice (post-internship)

**Organization of the new CRATB**

- Date of creation of the new CRATB
- Geographical location of the premises (detail the city and the host structure)
- Specialties represented
- Dedicated time in full-time equivalent (specify the FTE detail by specialty (general medicine, infectiology etc...)
- Non-medical time: secretary / nurse / other

**Articulation with the actors involved in the prevention of antibiotic resistance**

- Interaction with the Regional Health Agency: Y/N
- Frequency of meetings with the Regional Health Agency (number per year)
- Interaction with the regional IPC coordination center: Y/N
- Frequency of meetings with the regional IPC coordination center (number per year)
- Interaction with a local AMS consultant team: Y/N
- Frequency of meetings with local AMS consultant teams (number per year)
- Interaction with a local IPC consultant team: Y/N
- Frequency of meetings with local IPC consultant teams (number per year)
- Interaction with the hygiene or antibiotic therapy correspondents in the health centers or nursing homes: Y/N
- Frequency of meetings with hygiene/antibiotic therapy correspondents (number per year)
- Interaction with the university department of general medicine: Y/N
- Frequency of meetings with the university department of general medicine (number per year)
- Interaction with the National Health Insurance: Y/N
- Frequency of meetings with the National Health Insurance (number per year)
- Interaction with users: Y/N
- Frequency of meetings with users (number per year)
- Interaction with other CRATBs: Y/N
- Frequency of meetings with other CRATBs (number per year)

**Activities carried out**

- Training tools (for each tool, specify the sector of activity):
  - Training days / webinars / practical prescription assistance sheets / knowledge tests / tele-expertise / advice line
- Communication tools (for each tool, specify the activity sector):
  - Website / newsletter / social networks / smartphone application
- Among the three sectors of activity (hospitals, long-term care establishments and primary care), please specify WHICH one groups most of your actions
- Among the training and communication tools previously mentioned, please select the TWO that you consider the most crucial
- General public: Have you carried out any actions to raise awareness of AMS among the general public? Y/N
- AMS Indicators: Have you used AMS indicators? Y/N
- Activity Monitoring System: Do you have a system for tracking your activities? Y/N
- Practice audits / evaluation of actions taken? Y/N
- Have you set up research projects? Y/N

**Supplementary** Table S2 - Codebook for qualitative analysis

| **CODE** | **DEFINITION, INCLUSION/EXCLUSION, NOTES** | **CODE THEMES** |
| --- | --- | --- |
| **IMPLEMENTATION** | | |
| **Communication** | Nature, quality, and/or means of internal or external communications during implementation. *Exclude communications occurring after implementation.* |  |
| **Relationships** | Description of nature, quality, or temporality of relationships with internal or external actors. *Include descriptions of networks*. |  |
| **Planning** | Tasks or actions developed in advance related to implementation or future activities. |  |
| **Engaging** | Description of how key stakeholders were attracted and involved in CRATB implementation. |  |
| **Barriers** | Barriers to implementation at the regional level. |  |
| **Facilitators** | Factors facilitating implementation at the regional level. |  |
| **Expected benefits** | Perceptions about the benefits of CRATB implementation or comparisons to the current AMR prevention organization. |  |
| **CRATB ROLE** | | |
| **Actions** | Current and future activities implemented by the CRATB. |  |
| **Strengths** | Descriptions of perceived strengths within the organization or its programming. |  |
| **Weaknesses** | Descriptions of perceived weaknesses within the organization or its programming. |  |
| **INTERACTIONS WITH PARTNERS *(partners being defined as any organization or individual with whom potential collaborations exist, e.g., liberal professions (such as GPs, dentists, pharmacists,…), but also scientific societies, National Health Insurance, Long-term care centers, etc.)*** | | |
| **Communication** | Nature, quality, and/or means of communication between AMS actors. *Exclude communications relating to implementation process*. |  |
| **Barriers** | Barriers to partnerships and/or collaborations with regional AMS actors. *Exclude barriers related to implementation*. |  |
| **Facilitators** | Facilitators to partnerships and/or collaborations with regional AMS actors. *Exclude facilitators related to implementation*. |  |
| **Future perspectives** | Any future plans or ideas related to partnerships/collaborations. |  |

*AMS: antimicrobial stewardship; GP: General Practitioner*

**Supplementary Table S3 – Participant demographic data for the semi-structured** interviews.

| Gender M:F (Total) | Specialty/Profession |
| --- | --- |
| ***CRATB*** |  |
| 2:5 (7) | Infectious Disease specialist |
| 0:1 (1) | Hospital-based pharmacist |
| 1:0 (1) | IPC specialist |
| 0:1 (1) | Engineer |
| **3:7 (10)** | **Total CRATB participants** |
| ***Regional IPC coordination center*** | |
| 1:3 (4) | IPC specialist |
| 1:1 (2) | Public health specialist |
| 1:0 (1) | Clinical microbiologist |
| 1:0 (1) | Hospital-based pharmacist |
| 1:0 (1) | Infectious Disease specialist |
| **5:4 (9)** | **Total regional IPC coordination center participants** |
| ***Regional Health Agency*** | |
| 1:4 (5) | Physician |
| 0:3 (3) | Pharmacist |
| 0:1 (1) | Health executive |
| **1:8 (9)** | **Total Regional Health Agency participants** |
| **9:19 (28)** | **Total participants** |

Abbreviations: IPC: Infection Prevention and Control

**Supplementary Table S4 – Identified themes and illustrative quotations.**

Cross-cutting themes

| **THEME** | **RELATED CODE(S)** | **QUOTATIONS** |
| --- | --- | --- |
| T1- Streamlining and decompartmentalizing IPC and AMS activities  (7/8 regions) | Planning,  implementation facilitators,  CRATB strengths,  interaction facilitators | Subtheme: shared human and material resources  "They [the CRATB] have their offices, they aren't always there, but they have their offices in our office, so it is pretty easy [...]."  “We have the same offices, we have shared staff, shared secretarial support, we have a shared practitioner because we have a practitioner who is half-time [IPC coordinating center], half-time [CRATB]."  "So we have intersecting actions. [PX] is a member of [IPC coordinating center] and I'm a member [of the CRATB]. So we've crossed over. And then we have people like [PX] who is the typical example: [they are] part-time [CRATB] and part-time [IPC coordinating center]. So we try to interweave our participation as much as possible.”  "But it's true that since we [CRATB and IPC coordinating center] share the same offices as soon as they need specific data for a facility, we provide it to them." IPC coordinating center coordinator (Region 1)  "That is to say, she has two positions. 80% on the [IPC coordinating center] mission and 20% on the CRATB. But it isn’t a person who is within one entity who’s made available for the other. And the secretary is the same. The secretary is a full-time employee of the [IPC coordinating center] who, on an ad hoc basis, provides assistance to the CRATB. For example, for mailings, sending out documents. Things like that, providing mailing lists for emails, for communication.”  "So we have the mailing lists, we have [IPC coordinating center] data and data from other missions on [AMR] that are, that go through the [IPC coordinating center] and that are sent to the CRATB, for example." IPC coordinating center coordinator (Region 3)  "But actually we [CRATB and IPC coordinating center] have our offices in the same place. We have our offices, really close together. So it's pretty easy to interact.” CRATB coordinator (Region 4)  "We share staff, secretariat, webmaster, a bit of biostat, we have a signed agreement”. IPC coordinating center coordinator (Region 4)  "And otherwise, at this monthly meeting, there is [PX] and I who attend on the [IPC coordinating center] side, and there is also [PX] who is the data manager, who has been mutualized since January 1st."  "We shared the directory because when [the CRATB coordinator] arrived, so of course [they] had nothing in [their] inbox, in the computer, it was all empty [...] [They] had no mailing lists, nothing at all. So we contributed a lot. We gave everything we had in the way of mailing lists, whether it was the [AMS consultants] or the [Regional Health Agency] contacts. It wasn't always obvious at the beginning. We gave [them]a bit of background because it’s true that before the CRATB, there had been actions, there had been more or less optimistic attempted actions, one could say... " IPC coordinating center coordinator (Region 5)  "So no, my other activity is at the [IPC coordinating center] [...]." CRATB coordinator (Region 8)  "So we had the secretary who used to manage the mailings who did the secretarial work. And now, it's both [IPC coordinating center] but also CRATB. So it will... There will be something like, probably about 20% of [their] time, which will be in the spirit of mutualisation and synergy.”  "And in terms of resource persons who work in this structure, in fact, there is a doctor who is both a hygienist and an epidemiologist [...] who works part-time at the CRATB […] and part-time at the [IPC coordinating center]. So, it’s in fact through [them] that interactions are made easier.” IPC coordinating center coordinator (Region 8)  Subtheme: Effective collaboration  "And so I think that the [locally operating IPC consultant teams], when they are set up, and the [locally operating AMS consultant teams], will have every interest in working together since... it won't always be the same actors, but still Infectious Disease Specialists, coordinating physicians, GPs, pharmacists. Nurses eventually as well. So it is in our interest to work together to intervene in nursing homes and make things evolve in terms of [AMS] and infection prevention. So we have everything to gain by working together" CRATB coordinator (Region 1)  "So, as a result, a [IPC coordinating center]-CRATB team [...] went on site with the agreement of course of the establishment to work with the hygienists with the resource person on [AMS] in the establishment to try to improve prescribing practices." IPC coordinating center coordinator (Region 1)  "The [IPC coordinating center] and the [CRATB] have an extremely effective collaboration since they share offices, they share personnel, they share projects, they even share each other's governing bodies, so there is already more than just proximity." Regional Health Agency officer (Region 1)  "But with the territorial hygiene networks, we're really going to have a lot of support from them. And that's very interesting." CRATB coordinator 1 (Region 3)  "I What do you see as the greatest strengths of CRATB [Region 3] compared to other regions?  P The... Well, it's the structuring, and the fact that we work in synergy with the [locally operating AMS consultant teams] and other IPC coordinating center actors..." CRATB coordinator 2 (Region 3)  "But [CRATB officer X], he brings his expertise supporting the [IPC coordinating center]’s [AMS] missions. And so, communication happens by email almost...not daily, but sometimes several times a week."  "Or when there's a problem, for example, when we're asked to give advice. This happens to me from time to time in nursing homes. As soon as the situation drifts towards treatment, curative care, we hand over to the Infectious Disease Specialist... We hand over to the Infectious Disease Specialist because each of us has our own area of expertise. And that's it. I regularly call on our colleagues here [Hospital in city X], who are the [locally operating AMS consultant team], for advice on antibiotic therapy. That's pretty comfortable, actually." IPC coordinating center coordinator (Region 3)  "We are really, sincerely, the key words, it's really to decompartmentalize. We’re trying to decompartmentalize and to mutually call upon each other as soon as we do workshops, as soon as... even if they are fields that, *a priori*, do not directly concern either the [IPC coordinating center] or the CRATB."  "I am also an Infectious Disease Specialist, but in any case, it's obvious that this is a skill that must be present in a [IPC coordinating center], it's obvious. And so, if there were no infectious diseases time, I also believe that the proximity with the CRATB, therefore the access to infectious diseases competencies can only be beneficial for the [IPC coordinating center]. And conversely, the experience of the [IPC coordinating center] in operating as a network, in having contracts with the Regional Health Agency, etc., in keeping all this alive. This can only benefit the CRATB." IPC coordinating center coordinator (Region 4)  "Well, there are two circles. There's the [IPC coordinating center] agenda and the CRATB agenda. There's a common core to both actually. They connect on parts of their agendas."  "One last point that I may not have mentioned either is that in [Region 4], we have a coordinator for [AMR], well at the [Regional Health Agency] level. And a coordinator, which was initially called Healthcare Associated Infections, but which is now called Infection Prevention with the new strategy... different. We are two people in fact. It's about half-half in the regions. Some regions have chosen to have one person. But we're two. But we work together. So, knowing that there are not two of us working... So we already show this joint work of the two coordinators at the [Regional Health Agency].”  "In fact, the CRATB was created in September 2020, quite quickly in the [IPC coordinating center] offices. And since 2020 there is a joint action program." Regional Health Agency officer (Region 4)  "I would say that the strengths are the links that we have managed to create in one year with the [IPC coordinating center].”  "So it’s allowed us to breathe new life into a lot of projects that were underway. And to set up other... projects that we have in common with the [IPC coordinating center]. We've worked together a lot.” CRATB coordinator (Region 4)  "P And the last hope, well, the last very positive side, is that I also find that the collaboration with the [IPC coordinating center] is very productive, very pleasant and very efficient.  I It didn't exist before?  P Not so much. They have a much more structured perspective on what to do than an Infectious Disease Specialist. We can only benefit from that." CRATB coordinator 2 (Region 5)  "For example, the [IPC coordinating center], since it was required that the regional center work in close synergy with the [IPC coordinating center]. I based myself quite a bit on what they had, in particular they have a network that is already well developed. For example, the example that comes to mind is that I am working on the creation of a directory of antibiotic consultants in the region, which is complicated to set up, and the [IPC coordinating center] has very useful resources for this because they also have a directory. The first thing that comes to my mind is the collaboration with the [IPC coordinating center], which seems to me to be very important and maybe it's lucky for the region, compared to other countries. I don't know, but it's going really well." CRATB coordinator 1 (Region 5)  "We have [X number] of mobile hygiene teams that cover the region and it's clear that we need to ensure that they fit into this organizational chart for [AMS]. They are all quite enthusiastic about it, most of them are very, very keen on activities to promote [AMS] in nursing homes, which will probably be the key interface to reach the liberal sector.”  "So, these are perspectives, things on which we must work together with the CRATB to make any progress.” IPC coordinating center coordinator 2 (Region 5)  "I think that the purpose of the new strategy is co-sharing and co-construction. It's working in synergy and not... It's to decompartmentalize infection prevention and [AMR] prevention and work together. With all the local stakeholders.”  "It is very important because [AMS] cannot be dissociated from infections, in fact, from infection prevention. When, indeed, an action on [AMS] must also include the hygiene component, the diagnostic component... So it's true that we have to work differently and it has to be instinctive, if I may say so. And in fact, the organization that we want to put in place is designed to promote this sharing and this joint work.” Regional Health Agency officer (Region 6)  "But we have to rely on [IPC coordinating centers]. The [IPC coordinating centers] are totally used to this. They are much more... Some of them had already done a lot of things." CRATB coordinator (Region 7) |
| T2- Engaging with liberal health professions  (6/8 regions) | Engaging,  implementation barriers,  CRAbt strengths, CRATB weaknesses,  interaction facilitators, interaction barriers | Subtheme: Hiring GPs for CRATBs and locally operating AMS consultant teams  " We also tried to contact... Because in the [locally operating AMS consultant teams] there's an Infectious Disease Specialist, there's [a GP] too... So finding GPs who have time to get involved in [AMS], it's not easy. [...] but for the moment we haven't had any feedback. To try and start working with them, to try and have contacts and to build things. But for the moment we are struggling a bit, I have to say.” CRATB coordinator (Region 1)  "Because in fact, we have a General Practitioner. It's a mandatory part of the... I mean, it's part of the CRATB's minimum requirements, that is to say, at least one Infectious Disease Specialist and one General Practitioner. We had a hard time finding him, eh? It wasn't easy.” Regional Health Agency officer (Region 4)  "It was a hurdle [hiring a GP] for several months. Clearly, I spent a lot of time on it. It took... I contacted over a hundred GPs myself by getting them on the phone. Contacted them by email."  "So it was trying to see what was preventing us, to understand why we couldn't recruit. Basically, General Practitioners were interested in the project, but not in... they didn't have time to devote to it, dedicated time to spend on it, and they were afraid of the aspects of the regional mission related to the national ties. That's basically what was reported.” CRATB officer (Region 4)  “Frankly, I have already told [one of the national government representatives] that it’s very difficult to have a General Practitioner in a [locally operating AMS consultant team]... I mean in the CRATB, it's very difficult to find one..."IPC coordinating center coordinator (Region 4)  "I It's complicated in a lot of CRATBs to recruit a General Practitioner.  P1 Well yes, because we don't have golden bridges to offer them as...  P2 It's a bit of a strange activity for a GP to do anyway.” IPC coordinating center coordinators (Region 5)  "I And do you have any idea why it was difficult to motivate GPs to join this type of structure?  P Perhaps first of all, as the GP contact network wasn’t extremely complete, at least as far as I was concerned, perhaps the information wasn't circulated enough. And then, we have funding for a part-time GP. With the budget we had at the beginning, which was miserable, we had no chance of being able to convince a GP to join us part-time with the pay we were going to offer him. It took two months to be able to get a salary increase. It's just hard to recruit a part-time GP like that.” CRATB coordinator 1 (Region 5)  "And then there is a serious lack of GPs. To practice general medicine, for starters." CRATB coordinator 2 (Region 5)  "Oh no we don't have everything, since normally the CRATB should be composed of an Infectious Disease Specialist and a GP, so a large part of general medicine is missing. We've already published several ads on websites to try to get a motivated doctor. For the moment, it's not... It's a bit complicated, but we're going to try to start participating in certain training sessions for GPs to try, via the training sessions, to see if there are one or two who are interested.” CRATB coordinator (Region 7)  Subtheme: Communication and reach towards liberal professions  "I think we could do a better job with the nursing homes. And with the GPs, since GPs come to our seminars, for example, but it's always the same people and it's always those who feel concerned who come, so it's harder to reach the GPs who don't necessarily feel concerned.” CRATB coordinator (Region 1)  "With the [territorial health professional communities] as well, but the fact that we also recruited [CRATB GP1] who... He is a GP in [city X], and with [CRATB GP2] who is also a GP who is now [from the CRATB] who is a GP, they are 35-40 years old and they've set up a kind of group of GPs who are planning to do research in [AMS].” CRATB coordinator (Region 1)  "I don't know how many thousands of GPs we have in [region 3]. We don't have an easy entry point. It's very complicated. [...] Through the COVID crisis, we had some connections with the City, but I won’t lie to you, it's not great, the City. Why isn't it great? Because actually, the entry points are complex [...]." IPC coordinating center coordinator (Region 1)  "But really, we've been up against this from the beginning. Particularly with liberal physicians. How do you reach them when, in fact, you can't get their contact information? It's very difficult."  "I But with [the primary care sector], do you manage to have fluid and regular interactions or...  P No, no, no. Because the volume of people to be reached is too great. But we really need to be able to think about a large-scale project. We also have a lot of trouble getting lists to communicate this.”  "So, in order to have an impact on [primary care], it will be really interesting. In addition to having three [GP] colleagues who can help us... who can think and help us reach out to that sector." CRATB coordinator 1 (Region 3)  "But it's complex to work with liberal GPs for many reasons. The name "liberal" in itself is already one of the explanations. That is to say, since they're liberal, they do what they want. So that's one explanation. The second explanation is that they have lots of work with very tight schedules, and they don't necessarily want to have 30 minutes or even an hour in a consultation schedule to discuss other things. Which is that much less in terms of consultations. So yeah, liberal medicine is difficult. We have a lot of things to do, a lot of bridges to build. I don't really know exactly how we can do it." IPC coordinating center coordinator (Region 3)  "We don't have enough interaction with liberal GPs. Clearly. We have almost no interaction with dentists." IPC coordinating center coordinator (Region 4)  "I would say the strengths [...]. To have succeeded in recruiting a GP and in the objective of being able to develop [primary care]. Those are the two strong points for the first year.” CRATB coordinator (Region 4)  "But when we tried to reach prescribers in nursing homes as well, when we see the [MDROs]... But it's already harder because there can be 80 referring physicians when there are 80 residents."  "Where we have, I find, we have a lot of... a lot of room for improvement, is with respect to [primary care], [primary care] prescriptions. And that we haven't tackled because we have… it's difficult to reach them directly. We have to go through the [Regional Unions of Liberal Health Professionals]. And then, if we don't have specific information, we never get the mailing lists so we have to give them the information. And then if it works... Well, it's then distributed to all the liberal physicians, but if not, we can't." IPC coordinating center officer 1 (Region 5)  "Oh yes, you're right, liberal medicine is not easy. Knowing that it's there that we are expected to do a lot. And yes, one of the difficulties is how to reach out to liberal medicine as well.” CRATB coordinator 1 (Region 5)  "And our second hope is really to have a tool to act on [primary care] and to collaborate a little bit better with [the primary care sector]. We have a link, we have this hotline, so we've had a fan club of GPs for a long time. But on the other hand, we don't reach, or with great difficulty, a large part of GPs who don't ask for advice and work alone. Now I have hope that this will give us tools.” CRATB coordinator 2 (Region 5)  " And the other difficulty, which is a major one [...], is the involvement of GPs, prescribers and others. So veterinarians, maybe, we'll be able to reach them. They’re already sensitized. But perhaps we could reach even more dentists [...].  I Liberals in fact.  P Liberals, yeah. It's really going to be difficult I think. So that's really the fear I have, that it’ll remain... I mean, we're going to try to make sure from the start that we're not too hospital-oriented, but..."  "It's true that if we can't find a GP, if we can't reach them, if... there' s perhaps a point... The weakness is perhaps a difficulty in reaching the liberal world. By its [the CRATB] organization, already, from the outset by its organization in particular, carried by a university hospital, that' s perhaps its weakness.” CRATB coordinator (Region 7)  "And then, for the [Regional Unions of Liberal Health Professionals], it was associated with the creation of the [IPC coordinating center], normally of the CRATB, normally there’s a GP in it so I think that’s changing since [the GP] is having trouble following the meetings and all that. The connections...it's still a little bit difficult with all the liberal professions, the relationships." IPC coordinating center coordinator (Region 7)  "P And then, the fact that we've also rediscussed structuring, that we've kind of reset things with all the stakeholders and that we're participating with GPs. I think that's really good.  I You think that the creation of the CRATB will strengthen the links with GPs? Is that it?  P Yes. And then because we're going to work with them objectively, because we've found two. So we have two representatives...” CRATB coordinator (Region 8) |
| T3- Role of pre-existing networks and working relationships  (7/8 regions) | Planning, relationships, engaging, implementation facilitators,  implementation barriers,  CRATB strengths,  interaction facilitators | "The strong points I think is the history, since [the previous AMS structure] exists since 2003. So recognized for a while. I mean [the AMS network] is a network that is starting to be known in the region. So it gives some weight. And then the doctors call for telephone advice. They come for conferences, consult the website even if it's not... It could be better. But, so... There is still a history, a recognition at the regional level, collaborations with many infectious disease services and structures in the region.”  "Historically the [IPC coordinating center] [in Region 1] has been working with [the previous AMS structure] for a while. And [PX] and [PX] have been working together for a long time so the relationship is pretty good. I mean, they're good. With the [Regional Health Agency] as well.”  "I don't know all the actors yet because I arrived in the region two years ago, so I don't know everyone yet.” CRATB coordinator (Region 1)  "And then, there is networking with regional partners that was all set up, whether the [the Observatory for drugs, medical devices and therapeutic innovations] or the [Regional Medical Services Authority], since [AMR prevention] until now was really driven in a very energetic and effective way by the [National Health Insurance]" Regional Health Agency officer (Region 1)  "Because in [Region 2] we can almost say that the CRATB has existed informally for 10 years. What I mean is that we are really in contact with many stakeholders from different catchment areas and different specialties. So there is a certain organization that’s already there.”  "I think that it will be fairly simple if we start from the premise that there isn't really a team as defined by the decree yet, but that there are still quite a few teams in any case, of Infectious Disease Specialists, who in each establishment of the [Territory Hospital Group], [...] deal with infectiology." CRATB coordinator (Region 2)  "In any case, it's clear that they aren't in the same position as us in terms of history. Because you see, I've known all the hygienists and some of the nursing home managers for 20 years [...]. I know the managers, the regional doctors. I mean, we have a lot of contacts. They [the CRATB team] are mostly newcomers.” IPC coordinating center coordinator (Region 2)  "It's true that the people who are part of the [CRATB] have already been around for a while on these activities, we've already been working together for 3-4 years. It was quite easy and natural the... Our [Regional Health Agency] coordinating physician has also remained the same, so these teams are used to working together for several years now.”  "As I said in [Region 3], there were already actors involved in the matter. The [Regional Health Agency], but also the infectious diseases services, had already wanted to position themselves in a public health, [AMS] approach.”  "Well, its strengths, as I said earlier, is having existing resources, practitioners and... who know each other, local actors who have already worked together and projects that have already been developed and are running, particularly in terms of training. That, I think, is one of our great assets.” CRATB coordinator 1 (Region 3)  "It's true that we are lucky in the region to have an organization that already exists. Well, pre-existing actors. There was in fact... it wasn't nothing on the administrative level to bring all that together... But the actors were already established, so it was an asset.” Regional Health Agency officer (Region 3)  " Well, with the actors, it works well, especially with the National Health Insurance. That's particularly efficient. I mean, as soon as we need data from the National Health Insurance, the request is taken into account almost instantaneously. It's probably also a question of individuals. But there is a sensitivity to the issue on the National Health Insurance side. It's... there are links that have been established for a long time and it works incredibly well.” IPC coordinating center coordinator (Region 3)  "[Region 4] is a very agricultural region. [...] We already have ties with the [Regional Department of Agriculture, Food and Forestry] because two years ago... before COVID [...] we went to a meeting organized by the [Regional Department of Agriculture, Food and Forestry] for veterinarians, so... veterinarians and farmers. So the connections are made. We don't have any problems in [Region 4]. Historically, that's what we have as an advantage.”  "Yes, I think the pre-existing relationships have been important and you can see that we shouldn't expect all regions to operate at the same speed." Regional Health Agency officer (Region 4)  "But in fact, it was just resuming some of the connections that were already there.” CRATB coordinator (Region 4)  "We've already been working in the region with the establishment, under an experiment by the [Regional Health Agency], of a [Territory Hospital Group] [hospital practitioner]... in infectiology, who was responsible for coordinating the various [Territory Hospital Group] centers, for standardization, and for providing advice dedicated to those centers. In [city X], we had this history.”  "[...] we also have a history of working with [structure X] which is a structure that looks after the various hospitals in the [Territory Hospital Group] on the prevention of nosocomial infections. We've also worked a lot with them [...]. We've already benefited from their network and their organization. They introduced us to the big hospitals and that really saved us a lot of time."  "If we chose to offer the position to [PX], it was because we had actually worked with him in the context of the [French Infectious Disease Society] [AMS] reference group and in the context of our work of... around antibiotic therapy".  "The strengths of this structure [the CRATB] [...] it' s our history [...]. It's really a group of Infectious Disease Specialists who are used to working together and there are a lot of things that are rooted. In my opinion, that's a strength." CRATB coordinator 2 (Region 5)  "Well, a certain number of subjects were carried in work groups. So there's a dynamic that [a local AMS actor] had established long ago, I was going to say.” Regional Health Agency officer (Region 5)  "Before the CRATB, we were already working on [AMR] with Infectious Diseases Specialists and bacteriologists from the public and private sectors for hospitals. In general, I collected data, we conducted a survey and we were already working together. And so the CRATB was set up with these people with whom we already had quite a few exchanges and connections.” IPC coordinating center coordinator (Region 7)  "I had developed a lot, well I had developed the consults within my facility and also a little bit externally. And then, we participated with the [IPC coordinating center]...I mean, I participated quite a bit with the [IPC coordinating center] since 2017, in the creation of an advice line for the community, for infectious disease advice. It was with the creation of this advice line that I started to have the first connections with the [Regional Health Agency] and also with the [IPC coordinating center]." CRATB coordinator (Region 7)  "Knowing that I've already done training for [National Health Insurance] physicians, who go out and do peer visits, etc. We already have a connection, we've already been identified as a regional structure."  "I’m thinking of the [Observatory for drugs, medical devices and therapeutic innovations] when I say that, or the [local branch of the National Health Insurance]. We need to see people again, but it went really well the first time so there's no reason why it shouldn't continue to go well.”  "I really have a sense of unfinished business and that is the least we can say about the [local branch of the National Health Insurance], the [Regional Medical Services Authority], I think we will contact them very shortly to continue working with them.” CRATB coordinator 1 (Region 8)  Subtheme: Continuity of relationships and actions  " At this point, the [Regional Unions of Liberal Health Professionals] have the [AMS] project that we developed. We had liberal doctors, biologists, midwives, nurses, pharmacists [from the Regional Unions of Liberal Health Professionals]. So it was an inter-professional project. So I have contacts with these [Regional Unions of Liberal Health Professionals] '. [...] we continue to have interactions and the project continues." IPC coordinating center coordinator (Region 2)  "And then, above all, there was a willingness from the [Regional Health Agency] not to destroy what already existed. So, as a result, the antibiotic consultants and [structure X] we were already working together anyway. In fact, the CRATB was somewhat already created three years ago [...]. It was the logical continuation.”  "The CRATB is being established. And we change nothing. In fact, we are continuing the actions that are already in place. We will, we will, we won't change anything, we'll continue these actions and we'll especially develop new ones because we're better structured. CRATB coordinator 2 (Region 3)  " It' s the [Regional Health Agency] that' s in charge so.... And the [Regional Health Agency] sent out strong messages for the continuation of existing programming and organizations."  "So, as I was saying, we are also involved in a continuum.”  "It's not so much a feeling of implementation. It's a continuation of what we were doing and we have more time to do it.” CRATB coordinator 1 (Region 3)  "The role of the [Regional Health Agency] has been to coordinate, to achieve a more organized policy with the establishment of the regional strategic committee, the implementation of the CRATB, and finally to bring together the CRATB that already existed and to continue to have coordinated actions between [IPC coordinating center], [Observatories for drugs, medical devices and therapeutic innovations] and Infectious Disease Specialists." Regional Health Agency officer (Region 3)  "So, we had these two actors who were important in the region, knowing that [actor 1] won't disappear with the creation of the CRATB. They're still present. There's still funding from the [Regional Health Agency] to continue the [actor 1] adventure as well.” CRATB coordinator (Region 4)  "We have to build our regional organization in relation to the previous one. We're not going to say, let's just sweep up everything and start over and... that’s pointless."  "But we experienced this the day we had to tell the [Observatories for drugs, medical devices and therapeutic innovations] that they were no longer our support structure on the subject, just as we now have the CRATB. We have structures that have invested themselves for years and years and we can' t just replace them with a quick sweep. It's just not the right thing to do.”  "But in hospitals, we are just following ongoing actions, action that are already well established. Since 2002, in fact, since the first, the first plans. Regional Health Agency officer (Region 4)  "This is the principle that was pre-existing, so to speak, to the CRATB since [the previous structure] was already operating like that." Regional Health Agency officer (Region 6)  "Because now, we're thinking that the CRATB will promote a lot of the actions that we were carrying out before with [the previous AMS structure] in order to get our foot in the door.” CRATB coordinator (Region 8) |

Other themes related to CRATB implementation barriers and facilitators

| **Theme** | **Quotation** |
| --- | --- |
| *Code: Implementation barriers* | |
| Budgetary constraints | "And the [locally operating AMS consultant teams] also involve... Initially, the idea was one [locally operating AMS consultant team] per [Territory Hospital Group]. That means recruiting people, there is no dedicated funding for the moment. So it's not entirely simple. CRATB coordinator (Region 1)  "It's not necessarily going to be very easy. Yes, the [regional financing]. Because the GPs also have to be paid properly, for those who want to participate, otherwise they won't participate.”  "All of this adds up to a lot of money and I think everyone needs to understand that we need funding if we are going to get the ball rolling.” CRATB officer (Region 2)  "We hope to be able to launch the [locally operating AMS consultant teams] before the end of the year, in any case. But it's not simple, there are budget problems. Because until now, there was no dedicated funding to launch the [locally operating AMS consultant teams] and without money... It' s difficult to launch an [locally operating AMS consultant team].”  " Well, the idea is to set up the [locally operating AMS consultant team] with the Infectious Disease Specialist who is responsible for the team, but ideally associated with a pharmacist and a microbiologist, even if with the funding we have at present, it’s difficult to have really well-defined amounts of time for all these actors. Or else it would require much larger budgets than we have at present" CRATB coordinator (Region 4)  "It's super slow, I mean, I'll take an example like any other, which is money. That's always the crux of the issue. So, anyway. We had a budget that had been entrusted to us and then, following the revaluation of [financial] agreements and doctors' salaries, we needed a small increase in our budget. And so that we have to negotiate and it takes months and months." CRATB officer (Region 5)  "So you see the first discussions, we were supposed to have an answer around March, February, March and officially on the allocated budget, because the budget was going to somewhat determine what we were going to be able to implement. Of course. Well, we got the answer at the end of June. You see, there was a whole period where we really had the impression that we were already starting meetings, trying to start doing things, but without knowing exactly... So that was a bit of a pain. The fact that we didn't know what we were going to be able to do...”  "In particular, the issue of [locally operating AMS consultant teams] was quite complicated because in the text you had the CRATB for which there was funding but not so much for the [locally operating AMS consultant teams]." CRATB coordinator (Region 7) |
| COVID-19 | "In reality, the problem is that there was COVID in the meantime. You know, there were quite a few projects that were underway, but then they were stopped in the year of COVID.” CRATB coordinator (Region 4)  "It took us a while because there was COVID [...]. CRATB coordinator (Region 5)  "On the other hand, what really slowed down the process was the COVID context, of course. Obviously, it was a hindrance for everyone. But it's true that it really had an inhibiting effect because it weakened the connections between the teams, between the professionals. Regional Health Agency officer (Region 6)  "And then there was COVID. COVID set us back quite a bit.” CRATB coordinator (Region 7)  "In fact, it's fair to say that during COVID, discussions and substantive work were really put on the back burner completely." CRATB coordinator (Region 8) |
| *Code: Implementation facilitators* | |
| Stakeholder investment in AMS activities | "But apart from that, I think that it's in everyone's interest to really work together, to try to have common tools, to already use the tools that work and that exist as well as possible, to bring everyone into this effort. Personally, I feel very strongly about this. CRATB coordinator (Region 2)  " Oh yes. The actors are very involved.” Regional Health Agency officer (Region 3)  "And the other strength is our desire for [the CRATB] to work, since many of us expect it to help us in our practice.” CRATB coordinator (Region 5)  "Where [the Regional Health Agency director] said that [AMR prevention] should become a priority and he wanted things to move forward and for us to formalize our actions. He wants to hold a regional day for antibiotic consultants. So basically, he's in the... He's a driving force". IPC coordinating center coordinator (Region 6)  "And it's true that we have quite a few ideas. We have a lot of ideas.”  "So, I would say that the strength is [the CRATB’s] members and their drive. You know, its youth, I don't know how to put it. But for the moment its strength… its motivation and its members.” CRATB coordinator (Region 7)  "So, people are interested... After Covid, people want to reconnect, to see each other again, to do things together. So those we had already contacted previously, who already know us, are in fact already ready and they are very motivated."  "So we’ll probably work together again, [the local AMS actor] is very motivated to work with us again. That's good news too.” CRATB coordinator 1 (Region 8) |

Other themes related to perceived CRATB strengths and weaknesses

| **Theme** | **Quotation** |
| --- | --- |
| *Code: CRATB strengths* | |
| More effective AMS strategy and activities | Subtheme: collaborations  "But I think that working with Infectious Disease Specialists, the people who are in the institutions, who give directions for local policies in terms of [AMS], this could be a benefit to make the messages more relevant, targeted and help the health institutions to steer their [AMS] policies at the local level.” CRATB coordinator (Region 1)  "First of all, we’ll be working with teams that are neighbors, which is always very positive because we exchange a lot of things and then we progress, we move forward, we learn a certain number of things. I think it's also good to understand how things work at the regional level. Who are the key players, what are the levers, how can we use different tools to accomplish a number of things.” CRATB coordinator (Region 2)  "P1 [...] I hope that this will bring us closer to the [city 1] team and that we will be able to carry out larger-scale projects [...].  P2 [...] if you see the specialties of the Infectious Disease Specialists of [city 1] and [city 2], they are super complementary and not at all antagonistic. The themes are quite different, so there's no reason why there can't be collaborations.” CRATB coordinators (Region 5)  "The establishment of the CRATB, I think it' s an asset for us. Because it will redefine, reposition the governance and it will reposition all the work and it will revitalize the rest.” Regional Health Agency officer (Region 6)  "We had drawn up a roadmap, a roadmap on antibiotic resistance, in which we had described in the same document the actions of the [previous AMS structure], the [IPC COORDINATING CENTER] and the [Observatories for drugs, medical devices and therapeutic innovations], which were three actors who intervened and who sometimes intervened on somewhat similar themes and without necessarily much coordination.” CRATB coordinator (Region 8)  Subtheme: Time  "And then, on a very personal level, the fact that we became a CRATB, in concrete terms, I had a lot of trouble finding time to dedicate to the activity of antibiotic consultant with a clinical mission. My hygiene activity was a new addition, although it wasn't really part of my job description at the beginning. Well, now, I'm really going to be able to be positioned... to have a time, a part-time position completely dedicated to the CRATB, so it's really much more comfortable for me, to really be able to take on the mission and all the tasks that are involved and to be able to have a dedicated activity in this sense.” CRATB coordinator (Region 3)  "The first thing that is really, I think, is the fact of having been able to free up.... In any case, I'm only talking about our situation... It's the fact of having been able to free up someone to have a regional vision. In any case, for me, it's... it's things that in some cases, I was already doing without really having dedicated time for that, so keeping a clinical time. And here, to be able to say OK, I'm giving up my clinical time to really carry out this regional mission.” CRATB coordinator (Region 7)  "I have to admit that stopping the consults, even if it's something that works really well, it’s very, very time consuming and sometimes takes up all the room. So it takes up time in which you could be doing other things and background work. And so it's true that frequently when we had dedicated time for [the previous AMS structure] it was both consults and background actions and it was very difficult to free up some time. So that's really good.” CRATB coordinator (Region 8) |
| Team multidisciplinarity | "And we also hope that, you know, the [locally operating AMS consultant teams], in any case, the people who are going to work in the different [...] [locally operating AMS consultant teams], those that are being created, are quite vibrant people who I think are going to have projects at the departmental level as well, and as a result, that's going to create a bit of an emulsion too." CRATB coordinator (Region 3)  "[The CRATB officer] who is also a university professor, so doing a bit of research, so not bad. And the pharmacist is the pharmacist at [hospital X]. And so the other advantage we have is that we have a kind of territorial network, this CRATB.” CRATB coordinator (Region 7)  "As we said earlier, our strength lies in our distribution over the territory, the different... To tell you the truth, the leading trio is [CRATB member 1, CRATB member 2] and myself. In fact, we are complementary. [...] We don't have the same personalities. We are complementary in terms of skills and in terms of personalities.” CRATB coordinator (Region 8) |
| *Code: CRATB weaknesses* | |
| Geographical factors | "So, the weak points, well, we need to reinforce the regional coverage because the actions [of the CRATB] are very [city 1]-centric. This can be explained by the fact that the [previous AMS structure] in 200(?) was created where? Well in [city 1], with partners [from city 1]." Regional Health Agency officer (Region 1)  "There may be a problem. It's the fact that we are geographically distant, especially with [CRATB office 2]. Which can potentially impact communication flow. That's pretty logical. And that may be less the case here when we have people who are on this site, even if we are not in the same offices. We tend to think that the relationship is simpler when we are on site together, on the same site. So maybe the small downside is that some of the CRATB team are in [city 2], some are in [city 1]... So communication with the whole team isn't easy.” IPC coordinating center coordinator (Region 3)  "So effectively, [zone X of the region], we see that overall, there are a lot of things in place. In [zone Y of the region], there is quite a lot of inpatient care, but as P2 said, they do very little outpatient care. And then there are areas of the territory that are less dense in population. But there are areas where there are no Infectious Disease Specialists at all. The most striking thing that came out of the assessment we did is that there are really big disparities.” CRATB coordinator 1 (Region 5)  "I see weaknesses too. The territory is huge, it's really a big region. There are significant disparities in terms of population concentration, presence of Infectious Disease Specialists, which is what P1 told you.” CRATB coordinator 2 (Region 5)    "So I don't think we're going to have too much trouble rebuilding connections, even though there are areas that are still under-densified and under-populated in terms of physicians and consultants, which are [zone X] and [zone Y], which are two departments that are already poor in terms of GPs, poor in terms of hospital doctors and poor in terms of consultants and, of course, poor in terms of Infectious Disease Specialists. So, it's going to be complicated.” CRATB coordinator (Region 8) |

Other themes related to interaction barriers and facilitators between AMS actors

| **Theme** | **Quotation** |
| --- | --- |
| *Code: Interaction barriers* | |
| COVID-19 | "It's true that it's a bit of a lame excuse, but well, in this case, the lame excuse, yes, it's true that we've spent so much time with this COVID crisis that we've put aside a lot of other things." IPC COORDINATING CENTER coordinator (Region 1)  "I Of course, the COVID crisis has impacted things...  P But it's true that many work groups have unfortunately stopped, and projects haven't continued during this period.” Regional Health Agency officer (Region 2)  "Workshops for GPs, with COVID, have been somewhat abandoned [...].” CRATB coordinator (Region 3)  "We have the select committee, so we had a little interruption with COVID, no small thing..." Regional Health Agency officer (Region 3)  "That's a steering committee that normally took place, I think they did two or three a year. But then with COVID, we only had one in February.” CRATB coordinator (Region 4)  "It's a job that has been a bit... how would I say it, that has been a bit strained with the COVID crisis which has mobilized us heavily." Regional Health Agency officer (Region 5)  " After that, there was Covid right? It was just after Covid. I mean, Covid came right after. So there weren't really any collaborations." IPC coordinating center coordinator (Region 6)  "So we had a bit of a unique context, which is the context of COVID. So in all honesty, we had this initiative on the prevention of [AMR] that was put on hold in the sense that, in the end, the [AMS team] turned with all the regional actors to COVID and to the management of COVID." Regional Health Agency officer (Region 6)  "So that was stopped in COVID but we're going to restart them." IPC coordinating center coordinator (Region 7)  "We got cut off at the knees by COVID. We had a very interesting meeting in early February. We were going to do some work with, on dentists and on non-prescriptions and on the different drugs that the [local branch of the National Health Insurance] was looking at. But everything fell apart because of COVID.” CRATB coordinator 1 (Region 8)  "And this is a project that was effectively stopped because of COVID, but which may be resumed."  "So they invited us to be part of this group. But it didn't happen because of COVID.” CRATB coordinator 2 (Region 8)  "And then with COVID it was, we'll say, suspended on the one hand [...]." IPC coordinating center coordinator (Region 8) |
| *Code: Interaction facilitators* | |
| Shared resources (data and tools) | "On the other side [...] they've developed a lot of communication tools, especially YouTube channels, filmed staff meetings and things like that. So we can see that we have tools that can be interesting and complementary, but we’ll have to find a way to share the organization and the resources.” CRATB coordinator (Region 2)  "We have the National Health Insurance partner who is going to allow us to track some pretty specific indicators and see if it works. This will be a long-term project.” CRATB coordinator (Region 3)  "And we have an essential topic in our region, which is the evaluation of actions with the provision of indicators and statistics by the National Health Insurance.” Regional Health Agency officer (Region 6) |
| Individual characteristics | "The contacts I've had with [AMS actor 1] so far and with [AMS actor 2] are very nice, so I'd like to say that it can only be positive for us anyway" CRATB coordinator (Region 2)  "Then the human connections, as I was saying. If P1 and I didn't get along, we wouldn't be doing as many things either, that's obvious.” IPC coordinating center coordinator (Region 4)  " Then we also have a [Regional Health Agency] director, a [Regional Health Agency director general] who is very motivated and motivating. Maybe things will change now.” CPIAS coordinator (Region 6) |
| COVID-19 | "Oh yes, I discovered, I'm not going to lie to you, I discovered [welfare and care establishments] and amazing actors, fantastic people through the COVID crisis. People I didn't know.” CPIAS coordinator (Region 1)  "We do a face-to-face part for the restricted steering committees and a part that is accessible by videoconference which allows [City 1] and [City 2] to connect without having to travel. So that's a positive effect of COVID, which forced us to put all this in place.” Regional Health Agency officer (Region 3)  "P And we work a lot with videoconferencing now because of COVID.  I Yeah, a collateral effect...  P It' s had a booster effect.” CPIAS coordinator (Region 5)  "[...] the COVID crisis has strengthened our ties quite a bit.” Regional Health Agency officer (Region 5)  "In fact, Covid has allowed us to develop stronger ties with the [Regional Unions of Liberal Health Professionals].” CPIAS coordinator (Region 6) |

CRATB: Regional antimicrobial stewardship coordination centers; IPC: Infection Prevention and Control; AMS: Antimicrobial Stewardship; MDROs: Multi-drug resistance organisms
